# Mrs. Martha Weare Gratwick

**Published:** 1916-09

**Authors:** 


**Mrs. Martha Weare Gratwick,** widow of Wm. H. Gratwick, died in Buffalo July 26. She provided the funds for the erection of the Gratwick Laboratory, now included in the N. Y. State Institution for the Study of Malignant Diseases.

